# A Critical Review of the Status of Pesticide Exposure Management in Malawi

**DOI:** 10.3390/ijerph17186727

**Published:** 2020-09-15

**Authors:** Ishmael Kosamu, Chikumbusko Kaonga, Wells Utembe

**Affiliations:** 1 Department of Physics and Biochemical Sciences, The Polytechnic, University of Malawi, Blantyre, Malawi; ikosamu@poly.ac.mw (I.K.); ckaonga@poly.ac.mw (C.K.); 2 Toxicology and Biochemistry Department, National Institute for Occupational Health, Johannesburg 2000, South Africa

**Keywords:** pesticide, poisoning, contamination, food residues, obsolete pesticides

## Abstract

Pesticides pose a significant risk to humans and the environment. This paper analyzes the measures used to manage pesticides in Malawi. Malawi’s regulatory authority of pesticides, the Pesticides Control Board (PCB), faces a number of challenges including lack of facilities for analyzing pesticides and inadequate personnel to conduct risk assessment of pesticides. The PCB needs to provide access to information and opportunities among the public to make contributions regarding requirements, processes and policies for assessing pesticide risk and efficacy. There is also a need to enhance the capacity of PCB to assess pesticide poisoning in workers, monitor pesticide residues in food and environmental contamination, as well as to control the illegal importation and sale of pesticides. Just like in other countries such as South Africa, India and Sri Lanka, Malawi urgently needs to implement measures that can restrict the importation, production, sale and use of very toxic pesticides. Malawi also needs to develop measures for the effective management of pesticide waste containers as well as obsolete pesticides, where potential solutions include reducing the purchase of (unneeded) pesticides, treatment of obsolete pesticides in high-temperature cement kilns, as well as requesting pesticide dealers to adopt life-cycle management of their products.

## 1. Introduction

Pesticides include substances that are used to prevent, destroy, or control any pests in plants and animals, including humans. Pesticides have contributed to increased agricultural yields and food security [1,2] as well as more effective disease management around the world [3]. Specifically, the use of pesticides is crucial to the food security and economy of Malawi, where agriculture contributes 42 percent of Growth Domestic Product (GDP) and accounts for 81 percent of the foreign exchange earnings [4]. Malawi is located in Southern Africa, bordered by Mozambique to the east, south and part of the west, as well as Zambia to the west and Tanzania to the north. According to the Central Intelligence Agency (CIA) [5], Malawi has a total area of 118,484 km^2^ of which land covers 94,080 km^2^ while water covers 24,404 km^2^. The main economic activity is agriculture, which utilises 59.2% of the land (distributed as 38.2% arable land, 1.4% for permanent crops and 19.6% for permanent pasture). A further 34% of the land can be described as total forestry land, while the remaining 6.8% is used for other activities such as building construction and roads.

In Malawi, most of the pesticides are used in food and cash crops, with the usage ranking as follows: tobacco > tea > sugarcane > coffee > cotton > maize [6]. The major pesticides used in Malawi include Imidacloprid, Thiamethoxam, Carbaryl, Carbosulfan, Profenofos, Triazophos, Dimethoate, Cyfluthrin, Cypermethrin, Aldrin, Chlordane, Dieldrin, Heptachlor, Hexachlorobenzene, Mirex, Toxaphene, Chloropyrifos, Dichlorodiphenyltrichloroethane (DDT), Carboxin, Pirimiphos-methyl, Total permethrin, Deltamethrin, Fenitrothion, Atrazine, Metolachlor, Metryn, Acetochlor, Monosodium methylarsonate, Dichlorovos, Destab, Lambda cyhalothrin, Carbosulfan, Methyl parathion, Glyphosate, 2-4-D, Dicamba and Daconil [7,8].

Pesticides are also used in the control of many vector-borne diseases in Malawi. For example, insecticide treated nets (ITNs), indoor residual spraying (IRS) and other modes of pesticide applications are the most effective methods for the control of vector-borne diseases such as malaria [9,10].

Despite the numerous benefits in agriculture and public health, pesticides pose significant risks to humans and non-target organisms. For example, accidental or intentional acute pesticide poisoning continue to affect hundreds of thousands of people around the world [11], while many more also suffer from chronic effects of pesticide poisoning [12]. In this regard, although farmers in Africa use only 1–2% [13,14] of the world’s pesticides, they suffer most of the adverse effects because of a number of reasons that include unavailability of safety equipment, improper labeling of containers, illiteracy, ignorance and wrong attitudes [15]. Poor-quality and adulterated pesticides may render products ineffective or more hazardous which may result in over dosing [16]. The use of unregistered pesticides as well as very hazardous pesticides (World Health Organization (WHO) class I and II pesticides) has been reported in many countries including Nigeria [17], Ethiopia [18], South Africa [19] and Ghana [20]. Smallholder and commercial farmers in these countries utilize these pesticides in large amounts, with little regard to human health and the environment [20,21,22].

In order to avoid adverse effects of pesticides on humans and the environment, many countries, including Malawi, have adopted mechanisms for managing and controlling pesticides. These mechanisms include, inter alia, legislative and regulatory mechanisms, risk assessment and registration, testing of pesticide residues in food, as well as reporting and surveillance of pesticide poisoning cases. This paper analyzes the measures for the management of pesticides in Malawi and their implications on human health and the environment.

## 2. Methodology

This paper is a narrative review of literature retrieved from electronic searches of databases as well as hand searches of publications on pesticides in Malawi and other countries. The key words used in electronic searches on Google Scholar and PubMed include “pesticide” and “pesticide poisoning”. Furthermore, relevant information was sought from key informants through interviews. In this regard, individuals in key positions of relevant organizations were asked to provide insights on the involvement, mandate, successes and challenges of their respective organizations in pesticide management in Malawi.

## 3. Results

### 3.1. Pesticide Exposure and Poisoning

Pesticide poisoning can occur following acute or chronic (repeated) exposure. With the exceptions of pesticides such as warfarins, superwarfarins and coumarins that have delayed symptoms, an acute pesticide poisoning includes any health effect or illness that results from suspected or confirmed exposure to a pesticide occurring within 48 h [23]. Chronic poisoning, on the other hand, results from low exposure levels occurring over a significantly longer time. Pesticide poisoning is a major contributor to the global burden of disease, especially in developing countries [24]. Unfortunately, pesticide poisoning is often incorrectly regarded as a low public health priority area in many developing countries, where much emphasis is placed on infectious diseases.

Pesticide-related poisoning, with an estimated 168,000 deaths per annum, accounts for a significant proportion of 19.7% global suicides, while the proportion of pesticide-related suicides in Africa is 22.9% [25]. Easy access to pesticides in Malawi increases the potential for pesticide self-poisoning, where pesticides were involved in 79% of reported cases [26]. Furthermore, pesticides were reported to be among the leading causes of poisoning among children at the referral Queen Elizabeth Central Hospital in southern Malawi [27]. Admittedly, these studies are very old, but studies from other countries [28,29,30] indicate that pesticide-related illnesses still contribute significantly to poisoning cases.

Workers in developing countries are sometimes exposed to hazardous pesticides, including those that are severely restricted and banned in developed countries. For example, some pesticides such as ametryn, acetochlor, monosodium methylarsonate and profenofos are not approved in the European Union but are reported to be used on sugarcane farms in Malawi, where farmers were reported to suffer from skin irritation, headaches, coughing and running nose [31]. Unfortunately, there appears to be no comprehensive and systematic monitoring of occupational exposure to pesticides in Malawi.

The public in Malawi could be exposed to hazardous levels of pesticides through food, water, and residential use of pesticides. The use of pesticides in food crops could result in significant exposure to pesticides through food [32]. There have been reports of rather unusual risky practices among food-insecure communities of utilising pesticide-treated seeds for food consumption [33]. Although the extent of this practice is not known, it is important to investigate factors that lead to this practice as well as potential mitigation measures. In addition to this risky behaviour, there are concerns of acute pesticide poisoning following exposure to a number of pesticides with a common mechanism of action, especially for foods that are consumed in large amounts [34,35]. However, acute dietary pesticide poisoning is rare globally. On the other hand, the greatest concerns for pesticide exposure are associated with chronic exposure, especially since it has been shown that pesticide residues often exceed MRLs [36,37]. Furthermore, use of pesticides in food crops for which they were not authorized is also common [38]. Unfortunately, there appears to be no published studies conducted on pesticide residues in food in Malawi.

Pesticides can also contaminate surface and ground water through spray drift, surface runoff as well as drainage and percolation from treated areas. Cases of pesticide poisoning from drinking water are very rare [39], although pesticides have often been detected in drinking water [40,41]. Therefore, there are particular concerns for communities that utilize untreated drinking water from shallow wells located in agricultural areas. For example, atrazine and metolachlor were detected in surface water samples from Zomba and Bvumbwe areas in southern Malawi, especially with peak concentrations occurring in the first run-off event after pesticide application [42]. Exposure from spray drift, run-off and percolation is expected to be significant in large commercial farms that utilize large amounts of pesticides using aeroplanes, booms and tractors.

Since forestry areas occupy over 30% of the land, the improvement of the productivity of trees, plantations, and forest areas can be a significant source of environmental pollution. Pesticides are mostly utilised for controlling pests and weeds in forest renewal areas, especially in tree nurseries, but are rarely used in fully-grown plantations [43]. In the rare cases, Carbosulfan is used in the control of termites in miombo woodland forests to compliment “biopesticides” mainly made from of an extract of *Zahna africana*, ash and animal dung [44]. The use of the “biopesticides” is in line with the National Forest Policy [45] which aims to conserve, establish, protect and manage trees and forests for the sustainable development of Malawi, with minimal environmental pollution.

In addition to exposure to pesticides from agricultural use, pesticide poisoning can also occur through residential use of pesticides. For example, positive associations have been shown between residential exposures to unspecified pesticides during pregnancy and childhood and childhood leukemia [46]. Furthermore, household use of pyrethroid insecticides was shown to be a significant predictor of urinary pyrethroid metabolite levels in children [47]. Therefore, measures are needed to manage residential exposure to pesticides. However, there appears to be no studies on residential exposure to pesticides in Malawi.

Humans and other species can be exposed to mixtures of pesticides from various sources. Exposure to mixtures of pesticides is recognized as an issue of great concern since even though the exposure levels for each individual pesticide may be below thresholds of concerns, the combined effect may be significant [48].

### 3.2. Measures for Managing Pesticides in Malawi

#### 3.2.1. Legislation and Regulations on Pesticides in Malawi

The primary legal instrument for pesticide regulation in Malawi is the Pesticides Act [49]. The Act gives authority to the Pesticide Control Board (PCB) to control and manage the importation, exportation, manufacture, distribution, storage, disposal, sales, repackaging and use of all pesticides in Malawi. The PCB works in collaboration with other government agencies including the ministries responsible for health, environment, trade, and agriculture, through responsible government departments such as the Environmental Affairs Department (EAD), Malawi Bureau of Standards (MBS), the Environmental Health Services Division and the Department of Agricultural Research Services.

The Pesticide Act empowers the PCB to register agricultural pesticides and issue certificates and permits. The Act also prohibits the importation of any unregistered pesticide in Malawi. The Board shall only register pesticides that are suitable and effective for the intended use, and do not pose significant danger to human or animal health or the environment. Public health pesticides are registered by the Ministry of Health, through the Directorate of Epidemiology and Vector Control [6].

The Pesticides Act also empowers the responsible minister to prohibit or restrict the use of certain pesticides in any food products, and to establish maximum residual limits (MRLs), which are the maximum concentrations of a pesticide that are legally permitted in food commodities. The Pesticide Act operates in tandem with the Consumer Protection Act [50], which addresses issues concerning the rights of consumers.

The Environment Management Act (EMA, No 23 of 2017) [51] forbids any person from importing, exporting or transporting any hazardous waste or substance, except under a permit issued by the Minister. The Act also has provisions for monitoring of the effects of toxic and hazardous substances and their residues on human health and the environment, disposal of expired hazardous substances, and the restriction and banning of extremely toxic substances. The EMA is supported by the Water Resources Act (Controlled Water Areas) [52], which prohibits some activities, including the application of any pesticides in close proximity of water bodies.

In Malawi, the Occupational Safety, Health and Welfare Act (OSHW Act of 1997) [53] places the duty of care on every employer to ensure the safety, health and welfare at work for all employees, including the provision of protective clothing at no cost to the employee. In addition to the OSWHW Act, the Pesticide Act imposes the duty of care on employers regarding the use of pesticides at work as well as provision of “facilities, equipment and clothing conducive to the safe handling of the pesticide”. The Pesticide Act also empowers the registrar of pesticides to require “any employer to take steps to reduce the risks to the health of employees from pesticides, including requiring the employer to monitor the health of employees exposed to pesticides”. Furthermore, the Act requires notification to the registrar of pesticides of any pesticide-related incidents involving the death, illness or injury in humans (or wildlife).

In addition to the national pieces of legislation, Malawi is a signatory to international agreements including the Basel Convention on the control of transboundary movements and disposal of hazardous wastes [54], Rotterdam Convention on importation of hazardous chemicals [55], and Stockholm convention on persistent organic pollutants (POPs) [56].

#### 3.2.2. Pesticide Registration

Pesticide registration can be defined as a process through which a responsible national government authority approves the sale and use of a pesticide following comprehensive scientific evaluation or risk assessment [57]. According to the Acting Registrar of Pesticides, the PCB has many challenges including lack of analytical facilities for testing samples of candidate pesticides submitted for registration, inadequate human resources, and inadequate expertise for conducting risk assessment of pesticides as well as lack of clear guidelines for undertaking comprehensive risk assessment of pesticides [58]. Consequently, the PCB is not able to make sufficient inspections, offer sufficient training to key stakeholders and make sufficient tests to determine product quality [6]. The situation in Malawi is similar to other countries where the existence of weak governmental institutions results in the poor management of pesticides [59]. Therefore, there is need to enhance the capacity of PCB and other relevant agencies to manage pesticides in Malawi.

Risk assessment also results in the establishment of MRLs which should ideally be determined using generally acceptable practices (GAP) in locally produced agricultural commodities from local field trials covering the local range of climates, growing practices, crops, and cultivars [60]. Therefore, capacity is needed to allow the Minister of Agriculture to prepare MRLs based on local data, and not based on foreign data or on MRLs developed by the Codex Alimentarius Commission. The use of foreign data is not recommended since levels of residues in an agricultural commodity depend on prevailing agricultural practices and climate [61].

#### 3.2.3. Measures Limiting Access to Severely Hazardous Pesticides

Severely hazardous pesticide formulations (SHPFs) are defined in the Rotterdam convention as pesticides that are known to produce severe health or environmental effects observable within a short period of time after single or multiple exposure under conditions of use [62]. Additionally referred to as Highly Hazardous Pesticides (HHPs), these pesticides “present particularly high levels of acute or chronic hazards to health or environment according to internationally accepted classification systems such as WHO or Globally Harmonized System of Classification and Labelling of Chemicals (GHS)” [63] The majority of poisonings in Malawi have been attributed to the ready availability of these SHPFs or HHPs, especially the anticholinesterase organophosphates and carbamates [26]. Easy access to SHPFs (HHPs) has also resulted in many incidents of poisoning of protected wildlife [64,65]. Regulations that ban or restrict access to SHPFs or HHPs comprise one approach for reducing pesticide-related deaths in many countries [66,67,68].

However, there are questions regarding the effectiveness of this approach as a measure of reducing access to SHPFs or HHPs Malawi. For example, despite the banning of POPs in Malawi as a signatory to the Stockholm Convention of 2001, the illegal usage and selling of banned pesticides (such as aldrin, chlordane, dieldrin, and dichlorodiphenyltrichloroethane (DDT)) was shown to still be rampant in Malawi [8,69]. These pesticides are easily obtainable from unlicensed dealers and vendors, especially in the districts along the borders with Mozambique, Zambia and Tanzania [70]. Furthermore, the organophosphate Termik, which is registered for restricted use in the tea and coffee plantation, is readily available in markets [26]. Since these HHPs are banned by law but are still readily available, there is need to assess factors that contribute to their availability in Malawi. Moreover, Malawi should consider substituting WHO hazard Class I and II pesticides with safer and cost-effective alternatives.

#### 3.2.4. Pesticide Safety among Applicators and Pesticide Handlers

Knowledge, attitudes and practices of pesticide handlers have been shown in other countries to affect exposure to pesticides [71]. Unfortunately, very few studies have been conducted on knowledge, attitudes and practices of pesticide handlers and applicators in Malawi. While it could be shown in one study that most of the agro-dealers used personal protective equipment (PPE), not all local pesticides vendors used safety protection when selling pesticides [69]. It was also shown that the majority of sugarcane farmers did not use suitable PPE, with the exception of farmers that possessed tertiary education [31]. Therefore, there is need to intensify training on pesticide safety to pesticide handlers and farmers, together with provision of pesticide labels in vernacular languages [31]. Moreover, more studies on exposure to pesticides, levels of knowledge and attitudes towards pesticide safety and practices in pesticides handling are warranted for pesticide handlers in Malawi. Additionally, the few studies conducted in Malawi have indicated potential for significant occupational exposure to pesticides. Therefore, the comprehensive monitoring of occupational exposure is needed among pesticide handlers in Malawi.

#### 3.2.5. Post-Registration Monitoring and Surveillance

New pesticides may be shown to be reasonably safe in laboratory systems involving the use of models and a very restricted array of test species using a limited set of endpoints. It is important to note that the test organisms in the laboratory are not exposed to competition, predation and environmental fluctuations that are normally encountered in nature. The test organisms are also not exposed to natural complex chemical environment containing mixtures of potentially hazardous substances. For these reasons, there is a need for post-authorization pesticide monitoring, surveillance and toxicovigilance, activities that are designed to provide information on use patterns and impacts of each registered pesticide on public health and the environment [72].

Although the Pesticide Act requires notification to the registrar of pesticides of any such pesticide-related injury, this information appears not to be routinely, sufficiently and systematically collected in Malawi. This is consistent with studies from other countries which indicate serious underreporting [73]. Therefore, there is need for studies on adherence to this requirement and ways to reduce underreporting. There is also a paucity of data in Malawi on human and environmental exposure to pesticides as well as levels of knowledge, attitudes and practices (behaviors) related to pesticide handling and management.

As discussed earlier, there is also a lack of enforcement of pesticide-related legislation in Malawi. This inadequate control of pesticides has resulted in the availability of highly toxic pesticides for household use through informal vendors. These very toxic pesticides have been linked to numerous poisoning incidents that result from negligence, suicides and criminal acts in Malawi [26] and other countries [74,75]. Therefore, the surveillance of pesticides is needed in Malawi to monitor and assess use patterns and impacts of each registered pesticide on public health and the environment.

Although risk assessments conducted for pesticide registration can allow certain use patterns of a pesticide, the resulting residue levels can vary depending on application rates used, weather and climate as well as the type of crop. For these reasons, studies from other countries show that pesticide residues often exceed MRLs [36,76,77]. Furthermore, farmers have been reported to use pesticides that are not registered for application on a specific commodity [38]. For these reasons, many countries conduct food monitoring and surveillance programs to assess the levels of pollutants that various populations are exposed to [78]. Indeed, significant amounts of pesticide residues were determined in tomatoes that had previously been treated with pesticides in Malawi [79]. However, in Malawi, only agricultural commodities that are destined for the export market are routinely monitored for pesticide residues by the MBS at the request of the importing country. Therefore, Malawi needs to initiate routine food monitoring and surveillance programs to assess the levels of pesticide residues in food commodities consumed by the local population.

As discussed earlier, pesticides have often been detected in drinking water in Malawi. In this regard, different water treatment systems have been shown to have different efficiencies for removing pesticides from potable water supplies, where effectiveness of water purification depends on the type and dose (amount) of pesticides in the water [80]. The incidents of water contamination with pesticides indicate that water quality monitoring for pesticides is required in Malawi. Indeed, water quality monitoring is conducted by water boards in urban areas or by district assemblies in rural areas. However, these agencies do not have sufficient capacity to monitor the presence of a wide range of complex substances such as pesticides [81]. Therefore, there is need to increase the capacity for monitoring of pesticides not only in water but also other segments of the environment. The water utility companies in Malawi (Blantyre Water Board, Lilongwe Water Board, Northern Region Water Board, etc.) are encouraged to coordinate with other government agencies and universities that possess some capacity to analyze water for pesticides. For example, some analytical instruments, including High Performance Liquid Chromatography (HPLC), Liquid Chromatography–Mass Spectrometry (LC-MS) and Gas chromatography–Mass Spectrometry (GC-MS), can be found at various laboratories belonging to Malawi Bureau of Standards, University of Malawi, Bvumbwe Research Station, Chitedze Research Station, Forestry Research Institute of Malawi, and Pharmacy and Medicines Regulatory Authority, among others. Furthermore, some of these laboratories participate in national inter-laboratory quality management schemes, while the MBS oversees and participates in international and regional analytical instrument calibration programmes, mainly through the Southern African Development Community Accreditation Services (SADCAS).

Significant water contamination can also occur through spray drift [82]. For these reasons, many countries such as South Africa [83], Belgium [84], Netherlands [85], have instituted measures of preventing hazardous exposures from pesticide spray drift. However, Malawi does not yet have any measures or regulations that address pesticide drift.

In the use of pesticides in the forestry sector, Malawi would benefit significantly by joining forest management certification schemes, such as the Forest Stewardship Council (FSC) and the Programme for Endorsement of Forest Certification (PEFC). Forest certification is a voluntary process where an independent “certifier” assesses the quality of forest management and production against a set of requirements [86], which include use of pesticides in relation to risks to human health and the environment. As an example, the short-term objective of the FSC Pesticides Policy is to reduce the overall volume and number of chemical pesticides and eliminate the use of the most hazardous ones, while in the long-term, FSC aims to eliminate the use of chemical pesticides in all FSC-certified forests [87].

#### 3.2.6. Measures for Handling Pesticide Waste and Obsolete Pesticides in Malawi

Pesticide waste comprise of used pesticide containers, obsolete pesticides as well as wastewater from cleaning pesticide application equipment. Disposal of pesticide-related waste together with municipal waste can contaminate the environment. Furthermore, the use and recycling of empty or used pesticide containers for other purposes such as storage of food, feed, water and other items can result in significant exposure to hazardous pesticides [88]. It is estimated that 55,000 empty agricultural pesticide containers are produced annually in the central and southern regions of Malawi, representing 95% all empty containers generated in Malawi. Unfortunately, 70% of these containers are rinsed, punctured and stored on the original farms, while the rest are sold to pesticide resellers or recyclers and others are reused in households, where they pose significant risk to the general public [6]. Improperly disposed pesticide containers are reported to be used for packing some food products that are sold in markets and by road sides [69]. Therefore, there is a huge need for raising awareness among both pesticide distributors and the public about the health and environmental risks posed by used pesticide containers.

Unfortunately, Malawi lacks a comprehensive system for sustainably managing used pesticide containers. Currently, the Pesticide Act does not require any pesticide importers, formulators and retailers to adopt life-cycle management of their products, including empty containers, “from the cradle to the grave”. The Malawi standard on pesticide containers (MBS 89: 1991) stipulates that the containers should be disposed in a safe manner involving the removal of caps or lids as well as flattening, breaking, puncturing and burial in locations where there will be no pollution to ground or surface water [89]. An inventory conducted in 2012 showed that approximately 18,000 empty pesticide containers were stored in two central government locations in Lilongwe and Blantyre [6].

In addition to pesticide containers, stockpiles of obsolete pesticides can also be a source of exposure to humans and the environment. Pesticides become obsolete when they have lost their efficacy, and can no longer be used effectively for their intended purpose because of age, deterioration or a change of specified critical parameters or because they have been banned on account of their toxicity or environmental behavior [90]. Obsolete pesticides are often inadequately stored in corroded and leaking containers [91]. This often results in high levels of pesticide contamination in water and soil in areas where pesticides are stored.

The Food and Agriculture Organization (FAO) recommends four methods for the treatment of obsolete pesticides including high-temperature incineration, chemical treatment, use of engineered landfills, and long-term controlled storage [92]. High-temperature incineration is considered the most efficient option, but correct incinerators are not available in developing countries including Malawi. Therefore, obsolete pesticides are often transported to Europe for their destruction in dedicated high-temperature incinerators.

Malawi has accumulated an inventoried 382 tonnes of obsolete pesticide stocks attributed to centralized government procurement, poor stock management, inaccurate needs assessment, as well as weak import and regulatory controls. Some of these pesticides can be attributed to the centralized government procurement of large quantities of pesticides through the Farm Input Subsidy Programme (FISP) [6].

Since disposal or treatment of obsolete pesticides is more expensive than the purchase of stocks of pesticides, the best method of dealing with obsolete pesticides is by avoiding the purchase of unneeded pesticides. Obsolete pesticides can also be avoided or reduced through the practice of Integrated Pest Management Strategies (IPM). Furthermore, studies on the use of high-temperature cement kilns for the treatment of obsolete pesticides were shown in Korea and China to achieve destruction efficiencies of almost 100%, with negligible formation of other pollutants such as dioxins, furans and hexachlorobenzene [93,94]. Therefore, Malawi and other developing countries are urged to conduct economic and technical feasibility of using this option for the treatment of obsolete pesticides.

## 4. Conclusions

Measures used to manage pesticides in Malawi are summarized in Table 1 below.

This review shows that pesticides contribute significantly to acute poisoning cases in Malawi. On the other hand, the extent of chronic pesticide poisoning is not known because no such studies have been conducted. The review also reveals a lack of comprehensive and systematic monitoring of occupational and dietary exposure to pesticides in Malawi. Therefore, there is urgent need for these studies in Malawi. There is also a need to establish pesticide surveillance and monitoring systems in Malawi among pesticide handlers, in food and in water.

Malawi has some national and international legislative provisions conducive for the management of pesticides as well as the protection of workers, the public, and the environment. However, poor enforcement of legislation has resulted in rampant illegal vending of very toxic pesticides, which are linked to numerous poisoning incidents. Therefore, measures are urgently needed to minimize access to very toxic pesticides among the public. Unfortunately, the regulatory authority for pesticide management faces a number of challenges including lack of analytical facility for testing pesticides, inadequate personnel, inadequate expertise to conduct risk assessment of pesticides, and a lack of clear guidelines for undertaking comprehensive risk assessment of pesticides. Therefore, there is a need to enhance the capacity of PCB to manage pesticides in Malawi.

Malawi also needs to develop measures for the management of pesticide waste containers as well as obsolete pesticides. Potential solutions will include a reduction of the purchase of unneeded pesticides, use of high-temperature cement kilns for incineration of obsolete pesticides, as well as the amendment of the Pesticide Act to require any pesticide dealers to adopt life-cycle management of their products as well as empty containers.

## Figures and Tables

**Table 1:** Measures for pesticide management in Malawi in comparison to international best practice.

Aspect	Measures in Malawi	International Best Practice	Comment
Pesticide registration	Legal requirement in Malawi; however, risk assessment and MRLs based on foreign data; smuggling of unregistered pesticides across borders	Risk assessment and MRLs based on local data	Enhance the capacity of PCB to conduct risk assessment based on local data; strengthen control of unregistered pesticides along borders
Access to HHPs	Easy access to HHPs	Restricted access; registered vendors	Register pesticide vendors and dealers; criminalize sale of unregistered HHPs
Occupational exposure management	Handlers often handle pesticides without protection	Systematic use of PPE; registered applicators; surveillance of exposure among pesticide handlers	Intensify training on pesticide safety; provide pesticide labels in vernacular languages; register applicators
Pesticide poisoning surveillance	No systematic poisoning surveillance	Systematic poisoning surveillance	Make pesticide poisoning cases notifiable by law; develop comprehensive surveillance system
Pesticide residue testing in food	Routine testing of export commodities only	Routine testing of local agricultural produce	Enhance the capacity of the Ministry of Agriculture and MBS to conduct residue testing
Pesticides in water	Laws exist against water contamination with pesticides; However, there is no water monitoring for pesticides	Laws that protect water sources from contamination; routine water monitoring for pesticide contamination	Enhance the capacity of Water Boards to monitor for pesticides; prepare guidelines on spray drift management in large farms
Pesticides in the forestry sector	Encourage use of bio-pesticides	Subscription to Forest Management Certification such as FSC and PEFC	Encourage greater use of bio-pesticides and subscription to international forest management certification schemes
Pesticide waste and obsolete pesticides	Rampant reuse of pesticide waste, stockpiling of obsolete pesticides	Incineration and chemical treatment; proper management of pesticide waste containers	Amend the Pesticide Act to require pesticide manufacturers to adopt life-cycle management of their products; establish sustainable ways of handling obsolete pesticides

HHPs—highly hazardous pesticides; MRLs—maximum residual limits; PPE—personal protection equipment; FSC—Forest Stewardship Council; PCB—Pesticide Control Board; PEFC—Programme for Endorsement of Forest Certification.

## References

[1] Aktar W., Sengupta D., Chowdhury A. (2009). Impact of pesticides use in agriculture: Their benefits and hazards. Interdiscip. Toxicol..

[2] Cooper J., Dobson H. (2007). The benefits of pesticides to mankind and the environment. Crop Prot..

[3] WHO Pesticides and Their Application for the Control of Vectors and Pests of Public Health Importance. https://apps.who.int/iris/bitstream/handle/10665/69223/WHO_CDS_NTD_WHOPES_GCDPP_2006.1_eng.pdf.

[4] FAO Malawi. http://www.fao.org/3/y4632e/y4632e0n.htm.

[5] Central Intelligence Agency (CIA) (2020). The World Fact Book, Africa: Malawi. https://www.cia.gov/library/publications/the-world-factbook/geos/mi.html.

[6] Ministry of Agriculture, Irrigation and Water Development (2017). Pesticide Risk Reduction in Malawi (FSP). https://www.thegef.org/sites/default/files/project_documents/9-29-2014_ID5109__rev_reque_for_CEO_endorsment.pdf.

[7] Ministry of Agriculture, Malawi (1971). Cotton Handbook of Malawi.

[8] Soko J.J. (2018). Agricultural Pesticide Use in Malawi. J. Health Pollut..

[9] Mathanga D.P., Walker E.D., Wilson M.L., Ali D., Taylor T.E., Laufer M.K. (2012). Malaria control in Malawi: Current status and directions for the future. Acta Trop..

[10] Chanda E., Mzilahowa T., Chipwanya J., Mulenga S., Ali D., Troell P., Dodoli W., Govere J.M., Gimnig J. (2015). Preventing malaria transmission by indoor residual spraying in Malawi: Grappling with the challenge of uncertain sustainability. Malar. J..

[11] Dawson A.H., Eddleston M., Senarathna L., Mohamed F., Gawarammana I., Bowe S.J., Manuweera G., Buckley N.A. (2010). Acute Human Lethal Toxicity of Agricultural Pesticides: A Prospective Cohort Study. PLoS Med..

[12] Sánchez-Santed F., Colomina M.T., Herrero Hernández E. (2016). Organophosphate pesticide exposure and neurodegeneration. Cortex.

[13] Zhang W., Jiang F., Ou J. (2011). Global pesticide consumption and pollution: With China as a focus. Proc. Int. Acad. Ecol. Environ. Sci..

[14] Williamson S., Ball A., Pretty J. (2008). Trends in pesticide use and drivers for safer pest management in four African countries. Crop Prot..

[15] Kesavachandran C.N., Fareed M., Pathak M.K., Bihari V., Mathur N., Srivastava A.K., Whitacre D. (2009). Adverse Health Effects of Pesticides in Agrarian Populations of Developing Countries, in Reviews of Environmental Contamination and Toxicology.

[16] Brodesser J., Byron D.H., Cannavan A., Ferris I.G., Gross-Helmert K., Hendrichs J., Maestroni B.M., Unsworth J., Vaagt G., Zapata F. (2006). Pesticides in developing countries and the International Code of Conduct on the Distribution and the Use of Pesticides. Food Environ. Prot. Newsl..

[17] Oluwole O., Cheke R.A. (2009). Health and environmental impacts of pesticide use practices: A case study of farmers in Ekiti State, Nigeria. Int. J. Agric. Sustain..

[18] Kromhout H., Kromhout H., Mekonnen Y., Vermeulen R. (2016). Use of Chemical Pesticides in Ethiopia: A Cross-Sectional Comparative Study on Knowledge, Attitude and Practice of Farmers and Farm Workers in Three Farming Systems. Ann. Occup. Hyg..

[19] Naidoo S., London L., Burdorf A., Naidoo R.N., Kromhout H. (2008). Agricultural activities, pesticide use and occupational hazards among women working in small scale farming in Northern KwaZulu-Natal, South Africa. Int. J. Occup. Environ. Health.

[20] Denkyirah E.K., Okoffo E.D., Adu D.T., Aziz A.A., Ofori A., Denkyirah E.K l. (2016). Modeling Ghanaian cocoa farmers’ decision to use pesticide and frequency of application: The case of Brong Ahafo Region. SpringerPlus.

[21] De Bon H., Huat J., Parrot L., Sinzogan A., Martin T., Malezieux E., Vayssieres J.F. (2014). Pesticide risks from fruit and vegetable pest management by small farmers in sub-Saharan Africa. A review. Agron. Sustain. Dev..

[22] Asante K.A., Ntow W.J. (2009). Status of environmental contamination in Ghana, the perspective of a research scientist. Interdiscip. Stud. Environ. Chem..

[23] Thundiyil J.G., Stober J., Besbelli N., Pronczuk J. (2008). Acute pesticide poisoning: A proposed classification tool. Bull. World Health Organ..

[24] Prüss-Ustün A., Vickers C., Haefliger P., Bertollini R. (2011). Knowns and unknowns on burden of disease due to chemicals: A systematic review. Environ. Health.

[25] Gunnell D., Eddleston M., Phillips M.R., Konradsen F. (2007). The global distribution of fatal pesticide self-poisoning: Systematic review. BMC Public Health.

[26] Dzamalala C.P., Milner D.A., Liomba N.G. (2006). Suicide in Blantyre, Malawi (2000–2003). J. Clin. Forensic Med..

[27] Chibwana C., Mhango T., Molyneux E. (2001). Childhood poisoning at the Queen Elizabeth Central Hospital, Blantyre, Malawi. East Afr. Med J..

[28] Tattersall A., Clarke M., Marais S. (2008). Management and control of agri-chemical pesticides: Any progress?. Occup. Health South. Afr..

[29] Magauzi R., Mabaera B., Rusakaniko S., Chimusoro A., Ndlovu N., Tshimanga M., Shambira G., Chadambuka A., Gombe N. (2011). Health effects of agrochemicals among farm workers in commercial farms of Kwekwe district, Zimbabwe. Pan Afr. Med. J..

[30] Z’gambo J., Siulapwa Y., Michelo C. (2016). Pattern of acute poisoning at two urban referral hospitals in Lusaka, Zambia. BMC Emerg. Med..

[31] Donga T.K., Eklo O.M. (2018). Environmental load of pesticides used in conventional sugarcane production in Malawi. Crop Prot..

[32] Lu C., Barr D.B., Pearson M.A., Waller L.A. (2008). Dietary intake and its contribution to longitudinal organophosphorus pesticide exposure in urban/suburban children. Environ. Health Perspect..

[33] Schier J.G., Sejvar J.J., Lutterloh E., Likaka A., Katsoudas E., Karaseva Y.D., Barr B.T., Redwood Y., Monroe S. (2012). Consumption of pesticide-treated wheat seed by a rural population in Malawi. J. Expo. Sci. Environ. Epidemiol..

[34] Reeves W.R., McGuire M.K., Stokes M., Vicini J.L. (2019). Assessing the Safety of Pesticides in Food: How Current Regulations Protect Human Health. Adv. Nutr..

[35] Nicolopoulou-Stamati P., Maipas S., Kotampasi C., Stamatis P., Hens L. (2016). Chemical Pesticides and Human Health: The Urgent Need for a New Concept in Agriculture. Front. public health.

[36] Lozowicka B. (2015). Health risk for children and adults consuming apples with pesticide residue. Sci. Total Environ..

[37] Galani J., Houbraken M., Wumbei A., Djeugap J.F., Fotio D., Spanoghe P. (2018). Evaluation of 99 Pesticide Residues in Major Agricultural Products from the Western Highlands Zone of Cameroon Using QuEChERS Method Extraction and LC-MS/MS and GC-ECD Analyses. Foods.

[38] Jardim A.N.O., Mello D.C., Goes F.C.S., Junior E.F.F., Caldas E.D. (2014). Pesticide residues in cashew apple, guava, kaki and peach: GC–μECD, GC–FPD and LC–MS/MS multiresidue method validation, analysis and cumulative acute risk assessment. Food Chem..

[39] Panda M., Hutin Y.J., Ramachandran V., Murhekar M. (2009). A Fatal Waterborne Outbreak of Pesticide Poisoning Caused by Damaged Pipelines, Sindhikela, Bolangir, Orissa, India. J. Toxicol..

[40] Kuster M., de Alda M.J.L., Hernando M.D., Petrovic M., Martín-Alonso J., Barceló D. (2008). Analysis and occurrence of pharmaceuticals, estrogens, progestogens and polar pesticides in sewage treatment plant effluents, river water and drinking water in the Llobregat river basin (Barcelona, Spain). J. Hydrol..

[41] Donald D.B., Cessna A.J., Sverko E., Glozier N.E. (2007). Pesticides in surface drinking-water supplies of the northern Great Plains. Environ. Health Perspect..

[42] Lakudzala D.D. (2013). Atrazine and metolachlor contamination in surface and ground water in the Zomba/Bvumbwe region in Malawi. Int. Lett. Chem. Phys. Astron..

[43] Nthala N. (2020). Personal Communication.

[44] Kamoto J.F.M. (1999). Indigenous Silvicultural Practices of the Miombo Woodlands in Malawi: The Case of Five Villages Close to Chimaliro Forest Reserve. Community-Based Management of Miombo Woodlands in Malawi, Proceedings of the National Workshop, Sun and Sand Holiday Resort, Mangochi, Malawi, 27–29 September 1999.

[45] National Forestry Policy, Malawi. https://www.dof.gov.mw/storage/app/media/Policies%20and%20Strategies/National%20Forest%20Policy%202016.pdf.

[46] Turner M.C., Wigle D.T., Krewski D. (2009). Residential pesticides and childhood leukemia: A systematic review and meta-analysis. Environ. Health Perspect..

[47] Lu C., Barr D.B., Pearson M.A., Walker L.A., Bravo R. (2009). The attribution of urban and suburban children’s exposure to synthetic pyrethroid insecticides: A longitudinal assessment. J. Expo. Sci. Environ. Epidemiol..

[48] Sura S., Waiser M., Tumber V., Farenhorst A. (2012). Effects of herbicide mixture on microbial communities in prairie wetland ecosystems: A whole wetland approach. Sci. Total Environ..

[49] Government of Malawi (2000). Pesticide Act.

[50] Government of Malawi (2003). Consumer Protection Act.

[51] Government of Malawi (2017). Environment Management Act.

[52] Government of Malawi (2013). Water Resources Act (Controlled Water Areas).

[53] Government of Malawi (1997). Occupational Safety, Health and Welfare Act.

[54] Peiry K.K. (2013). The Basel Convention on the Control of Transboundary Movements of Hazardous Wastes and their Disposal: The Basel Convention at a Glance. Proceedings of the ASIL Annual Meeting.

[55] Jansen K., Dubois M. (2014). Global pesticide governance by disclosure: Prior informed consent and the Rotterdam convention. Transpar. Glob. Environ. Gov. Crit. Perspect..

[56] Fiedler H., Abad E., Van Bavel B., de Boer J., Bogdal C., Malisch R. (2013). The need for capacity building and first results for the Stockholm Convention Global Monitoring Plan. TrAC Trends Anal. Chem..

[57] WHO/FAO Guidelines for the Registration of Pesticides. https://www.who.int/whopes/resources/who_htm_ntd_whopes_2010.7/en/.

[58] Soko M. (2018). Personal comminatication.

[59] Stadlinger N., Mmochi A.J., Kumblad L. (2013). Weak governmental institutions impair the management of pesticide import and sales in Zanzibar. Ambio.

[60] Utembe W., Gulumian M. (2015). Challenges and research needs for risk assessment of pesticides for registration in Africa. Hum. Ecol. Risk Assess. Int. J..

[61] Handford C.E., Elliott C.T., Campbell K. (2015). A review of the global pesticide legislation and the scale of challenge in reaching the global harmonization of food safety standards. Integr. Environ. Assess. Manag..

[62] The Rotterdam Convention. http://www.pic.int/Procedures/SeverelyHazardousPesticideFormulations/tabid/1191/language/en-US/Default.aspx.

[63] WHO/FAO International Code of Conduct on Pesticide Management: Guidelines on Highly Hazardous Pesticides. http://www.fao.org/3/a-i5566e.pdf.

[64] Ogada D.L. (2014). The power of poison: Pesticide poisoning of Africa’s wildlife. Ann. N. Y. Acad. Sci..

[65] Roxburgh L., McDougall R. (2012). Vulture poisoning incidents and the status of vultures in Zambia and Malawi. Vulture News.

[66] Miller M., Bhalla K. (2010). An urgent need to restrict access to pesticides based on human lethality. Plos Med..

[67] Knipe D.W., Gunnell D., Eddleston M. (2017). Preventing deaths from pesticide self-poisoning—Learning from Sri Lanka’s success. Lancet Glob. Health.

[68] Gunnell D., Knipe D., Chang S.S., Pearson M., Konradsen F., Lee W.J., Eddleston M. (2017). Prevention of suicide with regulations aimed at restricting access to highly hazardous pesticides: A systematic review of the international evidence. Lancet Glob. Health.

[69] Makaya E., Tanyanyiwa V. (2016). Prevalence of Persistent Organic Pollutants in Blantyre—Malawi. Am. J. Environ. Prot..

[70] Ministry of Natural Resources, Energy and Environment National Profile to Assess the National Infrastructure for Management of Chemicals. http://cwm.unitar.org/national-profiles/publications/cw/np/np_pdf/Malawi_National_Profile.pdf.

[71] Oliveira Pasiani J., Torres P., Roniery Silva J., Diniz B.Z., Caldas E.D. (2012). Knowledge, attitudes, practices and biomonitoring of farmers and residents exposed to pesticides in Brazil. Int. J. Environ. Res. Public Health.

[72] Moss B. (2007). Water pollution by agriculture. Philos. Trans. R. Soc. B Biol. Sci..

[73] Rother H.-A. (2012). Improving poisoning diagnosis and surveillance of street pesticides. S. Afr. Med. J..

[74] Rother H.-A. (2010). Falling through the regulatory cracks: Street selling of pesticides and poisoning among urban youth in South Africa. Int. J. Occup. Environ. Health.

[75] Chang S.-S., Lu T.H., Eddleston M., Konradsen F., Sterne J.A., Lin J.J., Gunnell D. (2012). Factors associated with the decline in suicide by pesticide poisoning in Taiwan: A time trend analysis, 1987–2010. Clin. Toxicol..

[76] Osman K.A., Al-Humaid A.I., Al-Rehiayani S.M., Al-Redhaiman K.N. (2011). Estimated daily intake of pesticide residues exposure by vegetables grown in greenhouses in Al-Qassim region, Saudi Arabia. Food Control.

[77] Chen C., Qian Y., Chen Q., Tao C., Li C., Li Y. (2011). Evaluation of pesticide residues in fruits and vegetables from Xiamen, China. Food Control.

[78] EFSA, FAO, WHO (2011). State of the Art on Total Diet Studies Based on the Replies to the EFSA/FAO/WHO Questionnaire on National Total Diet Study Approaches.

[79] Mkandawire E. (2017). Evaluation of Traditional and Modernized Pest Control Methods Used by Smallholder Farmers in Malawi. Curr. Agric. Res. J..

[80] Ormad M.P., Miguel N., Claver A., Matesanz J.M., Ovelleiro J.L. (2008). Pesticides removal in the process of drinking water production. Chemosphere.

[81] Kayser G.L., Amjad U., Dalcanale F., Bartram J., Bentley M.E. (2015). Drinking water quality governance: A comparative case study of Brazil, Ecuador, and Malawi. Environ. Sci. Policy.

[82] Damalas C.A., Eleftherohorinos I.G. (2011). Pesticide exposure, safety issues, and risk assessment indicators. Int. J. Environ. Res. Public Health.

[83] South African National Standard The Aerial Application of Pesticides SANS 10118:2009. https://www.elaw.org/content/south-africa-sans-101182009-edition-3-south-african-national-standard-aerial-application-pes.

[84] De Schampheleire M., Baetens K., Nuyttens D., Spanoghe P. (2008). Spray drift measurements to evaluate the Belgian drift mitigation measures in field crops. Crop Prot..

[85] Van De Zande J., Michielsen J.M.G.P., Stallinga H., Wenneker M., Heijne B. Hedgerow filtration and barrier vegetation. Proceedings of the International Conference on Pesticide Application for Drift Management.

[86] FAO Forest Certification. http://www.fao.org/sustainable-forest-management/toolbox/modules/forest-certification/further-learning/en/?type=111#:~:text=Forest%20certification%20is%20a%20voluntary,public%20or%20private%20certification%20organization.

[87] FSC, FSC Pesticides Policy. https://www.fsc-uk.org/en-uk/business-area/fsc-certificate-types/forest-management-fm-certification/fsc-pesticides-policy.

[88] Damalas C.A., Koutroubas S.D. (2016). Farmers’ exposure to pesticides: Toxicity types and ways of prevention. Toxics.

[89] Malawi Bureau of Standards (MBS) (1991). The handling, Storage and Disposal of Pesticides and their Containers—Code of Practice (MBS 89: 1991).

[90] Obsolete Pesticides: A Ticking Time Bomb and Why We Have to Act Now. http://aei.pitt.edu/10965/1/1841%5B1%5D.pdf.

[91] Stockpiles of Obsolete Pesticides and Cleanup Priorities: A Methodology and Application for Tunisia. https://openknowledge.worldbank.org/handle/10986/5072.

[92] Felsot A.S., Racke K.D., Hamilton D.J., Ware G.W. (2003). Disposal and Degradation of Pesticide Waste, in Reviews of Environmental Contamination and Toxicology.

[93] Karstensen K.H., Kinh N.K., Viet P.H., Tuan N.D., Toi D.T., Hung N.H., Quan T.M., Hanh L.D., Thang D.H. (2006). Environmentally sound destruction of obsolete pesticides in developing countries using cement kilns. Environ. Sci. Policy.

[94] Li Y., Wang H., Zhang J., Wang J. (2012). Disposal of obsolete pesticides including DDT in a Chinese cement plant as blueprint for future environmentally sound co-processing of hazardous waste including POPS in the cement industry. Procedia Environ. Sci..

